# 

**Published:** 1875-03

**Authors:** 


### TABLE OF CONTENTS.

### ORIGINAL COMMUNICATIONS.

CLINICAL STUDIES with Large Non-Emetic Doses of Ipecacuanha: With a Contribution to the Therapeusis of Cholera. *By Alfred A. Woodhull, M.D., Asst. Surg. U. S. A., (Second Paper)* .......... 705

THE TREATMENT OF CONSTITUTIONAL OR TRUE SYPHILIS. *By Eugene Foster, M.D., Asst. Dem. Anat. Med. Col. of Georgia, Augusta, Ga* ...... 722

DIPHTHERIA. *By J. F. Cotten, M.D., of Acworth, Ga* .......... 731

RUPTURED PERINEUM. *By Robert Battey, M.D., of Atlanta, Ga* .......... 734

### ATLANTA ACADEMY OF MEDICINE.

Discussion upon Diphtheria and Scarlet Fever—Physiological Action of Mercury—Case of Ovariotomy—Therapeutical Effects of Electricity—Pistol Wound of Brain—Impaction of Fæces—Wine of Ipecac. in Atonic Dyspepsia and the Vomiting of Pregnancy .......... 738

### OUR EXCHANGES.

Sporadic Malignant Cholera .......... 749

The Management of Delirium .......... 750

Cerebro-Spinal Fever .......... 751

Absorption by the Skin .......... 752

On Catching Cold .......... 753

Cremation .......... 754

Vertigo as a Brain Symptom .......... 756

Bromide of Potassium in Hypertrophy of the Spleen .......... 757

An Odorless, Tasteless, Absolute Anti-Septic; Salicylic Acid .......... 758

Expedient in Difficult Labor .......... 759

Hereditary Vice .......... 759

Quinia—Its Therapeutical Action .......... 760

Whooping-Cough .......... 760

Diagnosis of Death .......... 760

Explanation of the Rigor in Puerperal Women Immediately after Birth of Child .......... 761

Ready Estimation of Fat in Milk .......... 761

Solution of Camphor in Erysipelas .......... 761

Excision of Three-Fourths of the Spleen .......... 762

### BIBLIOGRAPHICAL.

Atlee's Retrospect of Ovariotomy in Philadelphia .......... 762

Pamphlets .......... 764

### EDITORIAL.

State Board of Health .......... 764

Physicians' Fees .......... 764

Index .......... 765

